# Diabetes and Cognitive Decline: An Innovative Approach to Analyzing the Biophysical and Vibrational Properties of the Hippocampus

**DOI:** 10.1021/acsomega.4c05869

**Published:** 2024-09-19

**Authors:** Maria Do Socorro Do Nascimento Amorim, Erick Rafael Dias Rates, de Araujo Costa Melo Isabela Vitoria, Joel Félix Silva Diniz Filho, Clenilton Costa dos Santos, Ralph Santos-Oliveira, Renato Simões Gaspar, Jonas Rodrigues Sanches, Bruno Araújo Serra Pinto, Antonio Marcus de Andrade Paes, Luciana Magalhães Rebelo Alencar

**Affiliations:** † Federal University of Maranhão, Department of Physics, Laboratory of Biophysics and Nanosystems, Campus Bacanga, São Luís, Maranhão 65080-805, Brazil; ‡ Federal University of Maranhão, University School, Campus Bacanga, São Luís, Maranhão 65080-805, Brazil; § Brazilian Nuclear Energy Commission, Nuclear Engineering Institute, Rio de Janeiro 21941906, Brazil; ∥ Rio de Janeiro State University, Laboratory of Nanoradiopharmacy, Rio de Janeiro 23070200, Brazil; ⊥ Campinas State University, Translational Medicine Department, Campinas, Sao Paulo 13083888, Brazil; # Federal University of Maranhão, Department of Physiological Sciences, Laboratory of Experimental Physiology, Campus Bacanga, São Luís, Maranhão 65080-805, Brazil

## Abstract

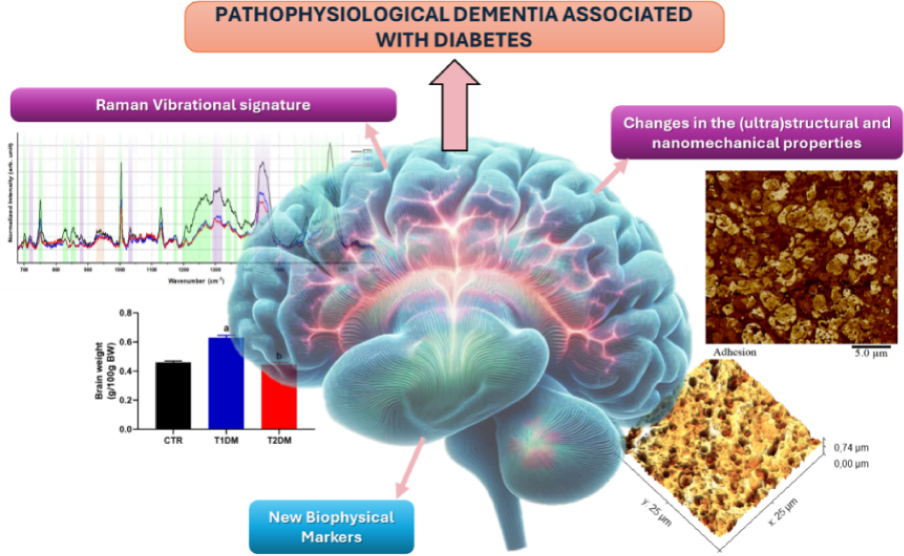

Diabetes Mellitus (DM) is a disease characterized by high blood glucose levels, known as hyperglycemia. Diabetes represents a risk factor for the development of neurodegenerative diseases, such as Alzheimer’s Disease (AD), one of the most prevalent neurodegenerative diseases worldwide, which leads to progressive mental, behavioral, and functional decline, affecting many brain structures, especially the hippocampus. Here, we aim to characterize the ultrastructural, nanomechanical, and vibrational changes in hyperglycemic hippocampal tissue using atomic force microscopy (AFM) and Raman spectroscopy. DM was induced in rats by streptozotocin injection (type 1) or dietary intervention (type 2). Cryosections of the hippocampus were prepared and analyzed on an MM8 AFM (Bruker) in Peak Force Quantitative Nanomechanics mode, performing 25 μm^2^ scans in 9 regions of 3 samples from each group. Ultrastructural and nanomechanical data such as surface roughness, area, volume, Young’s modulus, and adhesion were evaluated. The hippocampal samples were also analyzed on a T64000 Spectrometer (Horiba), using a laser λ = 632.8 nm, and for each sample, four spectra were obtained in different regions. AFM analyses show changes on the ultrastructural scale since diabetic animals had hippocampal tissue with greater roughness and volume. Meanwhile, diabetic tissues had decreased adhesion and Young’s modulus compared to control tissues. These were corroboratedby Raman data that shows changes in the molecular composition of diabetic tissues. The individual spectra show that the most significant changes are in the amide, cholesterol, and lipid bands. Overall, the data presented here show that hyperglycemia induces biophysical alterations in the hippocampal tissue of diabetic rats, providing novel biophysical and vibrational cues on the relationship between hyperglycemia and dementia.

## Introduction

Neurodegenerative diseases are progressive nervous system disorders that affect specific neuronal populations’ function and maintenance. The National Institute of Neurological Disorders and Stroke (NIH)^1^ describes more than 600 neurological disorders. It highlights Alzheimer’s and Parkinson’s diseases as the neurodegenerative diseases with the highest epidemiological incidence in the world.^2^ In 2020, it was estimated that 47 million people around the world were living with dementia, making it one of the main causes of dependence and disability. The number of people with dementia is predicted to increase in the next 30 years due to the population’s fast aging.^3^ Neurodegeneration is associated with synapse and neural network dysfunction and the deposition of physiochemically altered protein variants in the brain, such as tau and amyloid beta.^4^

Conversely, Diabetes mellitus (DM) is a chronic metabolic disease physiologically characterized by high blood glucose levels, mainly caused by insufficient insulin production or the body’s lack of insulin response.^5^ The International Diabetes Federation (IDF) estimates that 537 million adults (20–79 years) have diabetes and projects that by 2030, there will be 643 million, and by 2045, there will be 783 million.^6^

According to recent studies^7−9^ there is a correlation between diabetes and cognitive decline, with patients with diabetes being more predisposed to developing dementia than healthy individuals, particularly with many cases of Alzheimer’s Disease. Furthermore, statistical and biological evidence suggests a connection between dementia and diabetes^10,11^ possibly due to similar cellular and molecular pathways. Among various comorbidities, diabetes mellitus (DM) has the greatest influence on the development of Alzheimer’s Disease.^12^ Chronic hyperglycemia and microvascular disease also contribute to cognitive dysfunction in both type 1 and type 2 diabetes. Both types of diabetes are associated with mental and motor slowing, as well as similar reductions in measures of attention and executive functioning.^13,14^ Additionally, both types of diabetes are characterized by neural slowing, increased cortical atrophy, and microstructural abnormalities in white matter tracts.^15^

Alzheimer’s Disease could represent a specific form of Diabetes in the brain, which would be called Type 3 Diabetes Mellitus.^11,16−18^ In this context, there is growing attention on the role of β-amyloid and tau proteins in the peripheral nervous system and the induction of insulin resistance.^19^ Studies have shown that Alzheimer’s Disease and T2DM share common pathophysiological mechanisms associated with insulin resistance, such as oxidative stress, insulin signaling disorder, mitochondrial dysfunction, neuroinflammation, Advanced Glycation end Products (AGEs), and metabolic syndrome.^18,20^ The relationship between Diabetes and dementia is still poorly understood. A better understanding of the metabolic associations between DM and cognitive decline can provide a deeper understanding of the onset of diseases and the relationship between them, explaining, at least in part, their causalities. This understanding can be achieved with the help of experimental techniques that provide detailed information about the ultrastructure and biophysical and vibrational properties of biological systems, such as Atomic Force Microscopy (AFM) and Raman Spectroscopy.

AFM is a scanning probe technique developed by Gerd Binnig et al.;^21^ being a useful tool for nanoscale analysis due to the interatomic forces acting between the probe and the sample surface, ranging from 10 pN to 10 μN for small separations. Due to the long-range attractive forces, the vertical resolution of AFM is less than a nanometer, while the horizontal resolution is about 1 to 5 nm.^22^ The advantage of AFM over its predecessors is the possibility of studying conductive and insulating materials at ambient temperatures and biological systems under physiological conditions, such as tissues, cells, and viruses.^23−26^ For this reason, AFM has been widely used in research studying brain tissue.^27,28^

Raman spectroscopy is another versatile and nondestructive technique that analyzes the interaction between light and different systems. This technique was developed after studies by Indian physicist Chandrasekhar Venkata Raman and his student Krishnan.^29^ In Raman spectroscopy, a monochromatic light beam is directed at a sample, and the scattered light is collected and analyzed. Most of the scattered light has the same energy as the incident light, known as elastic scattering, but a small fraction of the light changes energy due to interaction with the molecular vibrations of the sample, known as inelastic scattering or Raman scattering.^30^ These energy changes, called Raman shifts, provide valuable information about molecular vibrations and chemical bonds in the sample, resulting in characteristic peaks that can be used to identify chemical compounds, determine molecular structure, and monitor chemical reactions in situ.^31^

Despite the applicability of these techniques and the relevance of cognitive impairment in DM, no studies address the relationship between hyperglycemia and dementia from a biophysical and vibrational point of view. This work presents an innovative approach to studying the correlation between hyperglycemia and cognitive decline. Data presented in the Supporting Information show that hyperglycemic animals have memory and learning decline using water maze and object recognition tests.

## Material and Methods

### Experimental Design

Wistar rats obtained from the animal facility at the Federal University of Maranhão were housed in a controlled environment (enriched with environmental stimuli, 22 ± 2 °C, humidity at 60%, 12-h light/dark cycle), provided with *ad libitum* access to water and standard chow. All animal procedures were approved by the Ethical Committee on Animal Use and Welfare of the Federal University of Maranhão, under ruling number 23 115.000747/2022–90.

Figure S1 includes a timeline graph describing the diets and interventions used, which helps to better understand the experiments. Female Wistar rats (*n* = 6; 21 days old) were categorized by weight into two groups: rats fed a standard chow (Nuvital, Nuvilab, Brazil) and rats fed a high-sucrose diet (HSD) to induce type 2 diabetes mellitus (T2DM) in their offspring. As previously described, HSD was manufactured by adding the standard diet with condensed milk and refined sugar.^32^ After 6 weeks of monitoring, the females were housed individually for monogamous mating, and the males were fed exclusively with standard chow. Following confirmation of pregnancy, the females were separated from the males and kept with the offspring until weaning.

Maternal exposure to obesogenic diets, particularly those high in saturated fats and added sugars, during gestation and lactation has been increasingly linked to the development of type 2 diabetes mellitus (T2DM) in offspring.^33^ This association is rooted in the Developmental Origins of Health and Disease (DOHaD) concept, which posits that environmental factors during critical periods of early development can have lasting impacts on an individual’s health.^32,33^ The literature consistently shows the rodent model of metabolic syndrome induced by high sucrose diet (HSD) to study the late-in-life harmful metabolic effects of early in-life exposure to simple sugars.^34−37^

After weaning, the male offspring were segregated and allocated into three groups as follows: control group (CTR; *n* = 5), consisting of offspring from control dams that did not undergo any intervention for 24 weeks; type 1 diabetes mellitus group (T1DM; *n* = 5), comprising offspring from control dams who, at the 12th week of life, received a single dose of streptozotocin (65 mg/kg; i.p.; citrate buffer; pH 4.5; after 12-h fasting)^38^ and were then maintained for another 12 weeks; and finally, type 2 diabetes mellitus group (T2DM; *n* = 5), comprising offspring from mothers who received HSD before and during pregnancy to induce T2DM but were exclusively fed standard chow for 24 weeks.

Throughout the entire postinduction follow-up period (12 weeks), weekly assessments of body weight were conducted. In the 11th week, animals underwent an 8-h fasting period before administering 2 g/kg of intraperitoneal glucose to assess glucose tolerance (GTT test). Tail vein blood samples were collected immediately before (time 0) and 15, 30, 60, and 120 min after the glucose bolus for glucose measurement using a glucometer (Accu-check Active, Roche). The data are expressed as the area under the curve of glycemic levels.^37^ Also, in the 11th week, the groups underwent cognitive tests—novel object recognition^39^ and water maze.^40^ Details of the cognitive and diabetes tests and their results are available in the Supporting Information.

Finally, in the 12th week, the animals were fasted for 8 h, anesthetized, and had their nasal-anal lengths measured to assess obesity using the Lee index.^41^ Subsequently, they were euthanized by puncturing the descending aorta and underwent laparotomy for the collection of retroperitoneal, periepididymal, and mesenteric fat pads. Additionally, the animals underwent a craniotomy to remove the brain and isolate the hippocampal tissue.

The collected blood was allowed to clot and then centrifuged (3500 rpm, 10 min) to separate the serum, which was used to determine glucose and triglyceride levels (Labtest, Brazil) and insulin levels (Sigma-Aldrich, Germany) according to the specifications provided by the kit manufacturer. From these dosages, insulin resistance was determined using the HOMA-IR.^42,43^ The collected organs were weighed for morphometric evaluation, while the hippocampi were dissected for subsequent assessments.

### Sample Preparation

After euthanasia, the brains were rapidly removed and placed in liquid nitrogen to minimize tissue degradation. This process preserves the native tissue structure without introducing chemical fixatives that may alter the tissue’s mechanical properties. Sections were prepared using a cryostat, which allows for the cutting of frozen samples into ultrathin sections (typically between 10 and 50 μm thick), as employed by other authors.^44−46^ The cryostat temperature was maintained between −20 °C and −30 °C to ensure precise cuts and avoid tissue deformation. Tissue sections were mounted on glass slides for AFM and Raman Spectroscopy analysis. The slides with tissue sections were air-dried at room temperature. All these steps ensure the highest tissue preservation, providing greater reliability and fidelity in quantitative data, as minimal intervention occurred.

### AFM Setup

Atomic Force Microscopy was analyzed using an AFM Multimode 8 (Bruker, Santa Barbara, CA, USA) in PeakForce Quantitative Nanomechanics (QNM) mode. qp-HBC (Nanosensors) probes with a nominal spring constant of 0.5 N/m and a tip radius of <10 nm were used for all measurements. All data acquisition was done in an air environment (23 °C temperature and 44% humidity) with scan parameters of 0.3 Hz scan rate and 25 × 25 μm^2^ scan area for tissue analysis. Each sample group had three hippocampal sections, and nine regions were analyzed for each hippocampal section, totaling 27 maps per sample group. The regions analyzed comprised CA2 and CA3 of the hippocampus.

The Peak Force Quantitative Nanomechanics (PF-QNM) mode in atomic force microscopy enables detailed nanoscale characterization of the mechanical properties of materials, including stiffness, adhesion, and energy dissipation.^47^ During scanning, the AFM tip makes periodic contact with the sample surface. In each contact cycle, the tip approaches the surface, applies increasing force until it reaches a maximum value (peak force), and then withdraws, recording a force–distance curve at each contact. This curve measures the force as a function of the distance between the tip and the surface, providing insights into the nanomechanical properties of the sample. The force curve is essential for analyzing surface characteristics and can be measured off-resonance or close to the fundamental resonance of the cantilever.^48^

### Raman Setup

A triple Raman spectrometer (model T64000, Horiba) was used, operating in the single model with a resolution of less than 2 cm^–1^. The instrument has a liquid N_2_-cooled charge-coupled device (CCD) detector. 532.0 nm green light (LAS-532–100-HREV) operating at 14 mW was employed for excitation. A long working distance objective lens (100x, 18 mm) was used. The spectra were acquired at five different points on the surface for each sample group after five acquisitions of 30s each in each range of the degree of spectral dispersion. As for AFM measurements, the regions analyzed comprised CA2 and CA3 of the hippocampus, always maintaining the region analyzed for each sample, with prior visualization under an optical microscope.

### Data Analysis

For analysis of statistical roughness data, the chosen parameter consists of the height of each pixel of the height maps through the mean quadratic roughness R_q_, as it represents a standard deviation of the distribution of heights on the surface, according to the methodology used by Rates and collaborators.^49^ In summary, before the roughness analysis, the maps were pretreated with third-order polynomial adjustment, allowing more significant height differences, especially promoted by sample preparation, to be minimized, resulting in topography with more substantial contributions from the structures of the hippocampus surface. Topographic maps measuring 25 × 25 μm^2^ in area were analyzed at this stage. Nanomechanical adhesion and Young’s modulus data were calculated from force curves obtained through force spectroscopy experiments. Young’s modulus data could be extracted from fits to the force curves over a deflection range. Using the model of a cone-shaped indenter, the Derjaguin Muller Toporov (DMT)^50^ considers the adhesion forces. According to Cardoso-Lima and coworkers’ methodology,^51^ the adhesion force was calculated from the retraction curves, considering the minimum value of cantilever deflection. This value represents the probe’s resistance to leaving the surface of the hippocampal tissue. The tissue surface area is computed by simple surface triangulation using statistical analysis from the software Gwyddion 2.60.^52^ The tissue volume was calculated as the integral of the surface height over the scanned area using the same software.^52^

Raman spectroscopy data processing was performed using LabSpec6 software. The narrow peaks caused by cosmic rays were removed in sequence, and the fluorescence background variation and the glass substrate were estimated using the fifth-order polynomial fit and subtracted. The Principal Component Analysis (PCA) technique was applied to the spectral data set, using a statistical method capable of reducing the dimensionality of the data at the same time as the response of most of the variation present in the original data. The analysis of variance is used on the scores of the ten main components to identify which main components present significant differences in the mean scores between the two groups of cells.^53^ This analysis was conducted using OriginLab software.

### Statistical Analysis

Statistical analysis was conducted using GraphPad Prism 8.0 software (GraphPad Software Inc., USA) and Origin 2024 software (Originlab Inc., USA). Data from animal tests were expressed as mean ± SEM and submitted to a normality test (Kolmogorov–Smirnov) followed by one-way ANOVA (posttest Tukey) for a significance level of 5% (*p* < 0.05).^54^ We have a value associated with each force curve for nanomechanical data involving adhesion and Young’s modulus, totaling 65 536 force curves per map. For roughness data, a single Rq value is acquired per map, considering each image pixel in height (256 × 256 pixels). The calculated error was the standard deviation (SD) across all data.

## Results

### Ultrastructural Results of Hippocampus Analysis

**Figure 1:**
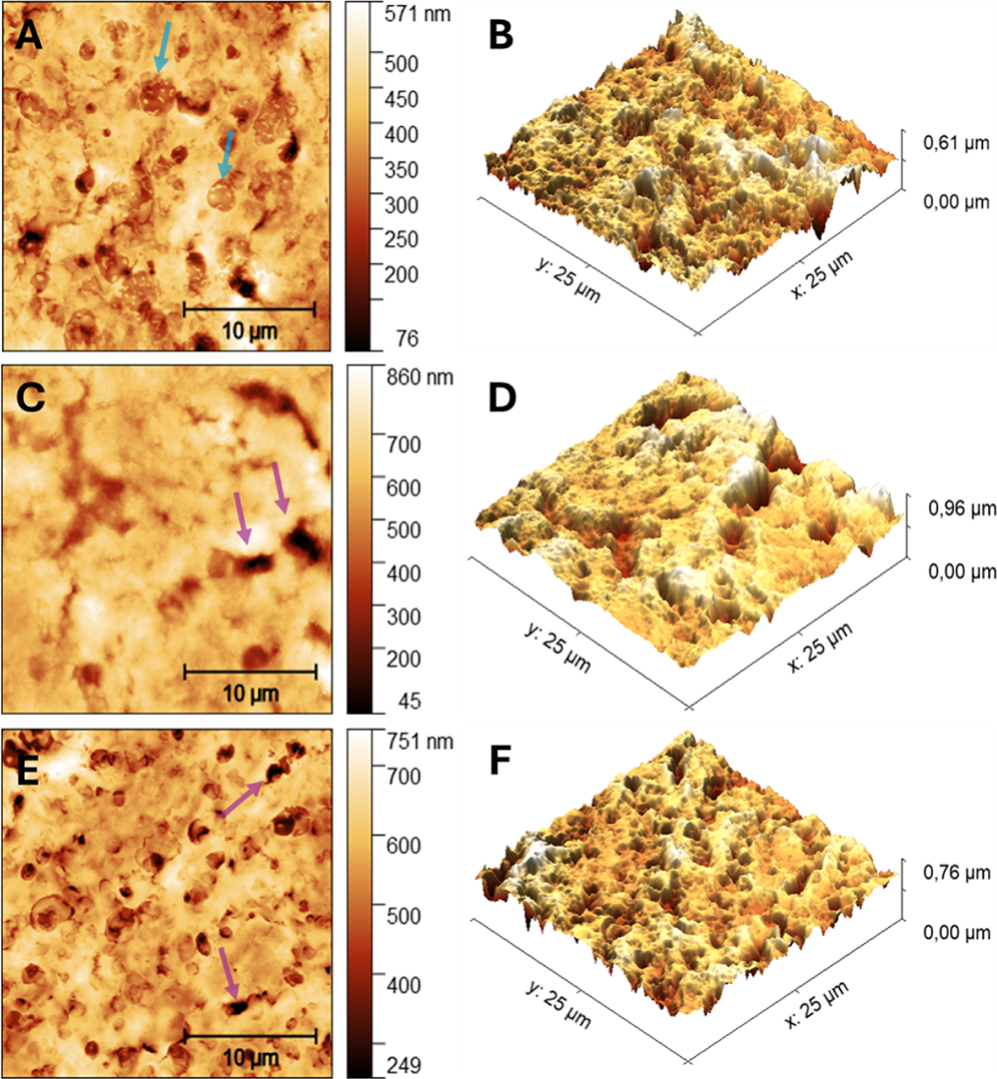
AFM morphological maps. **A.** Height map of the control hippocampal tissue. Blue arrows point to structures observed only in control hippocampal tissue. **C.** Height map of the tissue of the T1DM and **E.** T2DM group. Purple arrows point to holes in T1DM and T2DM tissue. Figures **B**, **D**, and **F** show their respective three-dimensional maps.

To assess whether the cognitive decline of T1DM and T2DM rats was associated with biophysical changes, we used AFM to study the morphological features of their hippocampus. The morphological study of brain tissues through techniques such as AFM is of great importance as it allows visualization of structures at the nanoscale, providing precise details of brain tissue morphology, which can be essential for understanding the microscopic organization of the brain and identifying structural alterations associated with neuropathological conditions. These findings can help understand the underlying mechanisms of these diseases and identify biomarkers.^28,55^Figure 1 shows differences in the morphology of TDM tissues compared to control. First, when looking at the height scale bar, it is seen that this scale increases for the TDM groups (Figure 1C,D and E,F) compared with the control group (Figure 1A,B), with emphasis on the sample with T1DM, indicating greater differences in height, possibly associated with the holes observed in these tissues (purple arrows). In the morphological profile of the control group, structures internal to the pores are present that are not seen in the samples from the TDM groups (blue arrows).

In Figure 1A,C,E, several holes can be seen in the morphological profile of the samples. Using Gwyddion Software, the mean diameter and depth of the holes (*n* = 30) of each representative map for the samples were quantified with standard error. The results of the hole diameters for the control, T1DM, and T2DM samples were, respectively, 1.4 ± 0.1 μm, 3.1 ± 0.3, and 2.6 ± 0.1 μm. For the depth calculation, the results for the control, DM1, and DM2 samples were 178.8 ± 27.6 nm, 513.1 ± 52.9, and 453.7 ± 32.3. These data indicate a significant increase in the size and depth of holes in DM1 and DM2 samples compared to control samples, suggesting greater tissue damage in diabetes cases.

**Figure 2:**
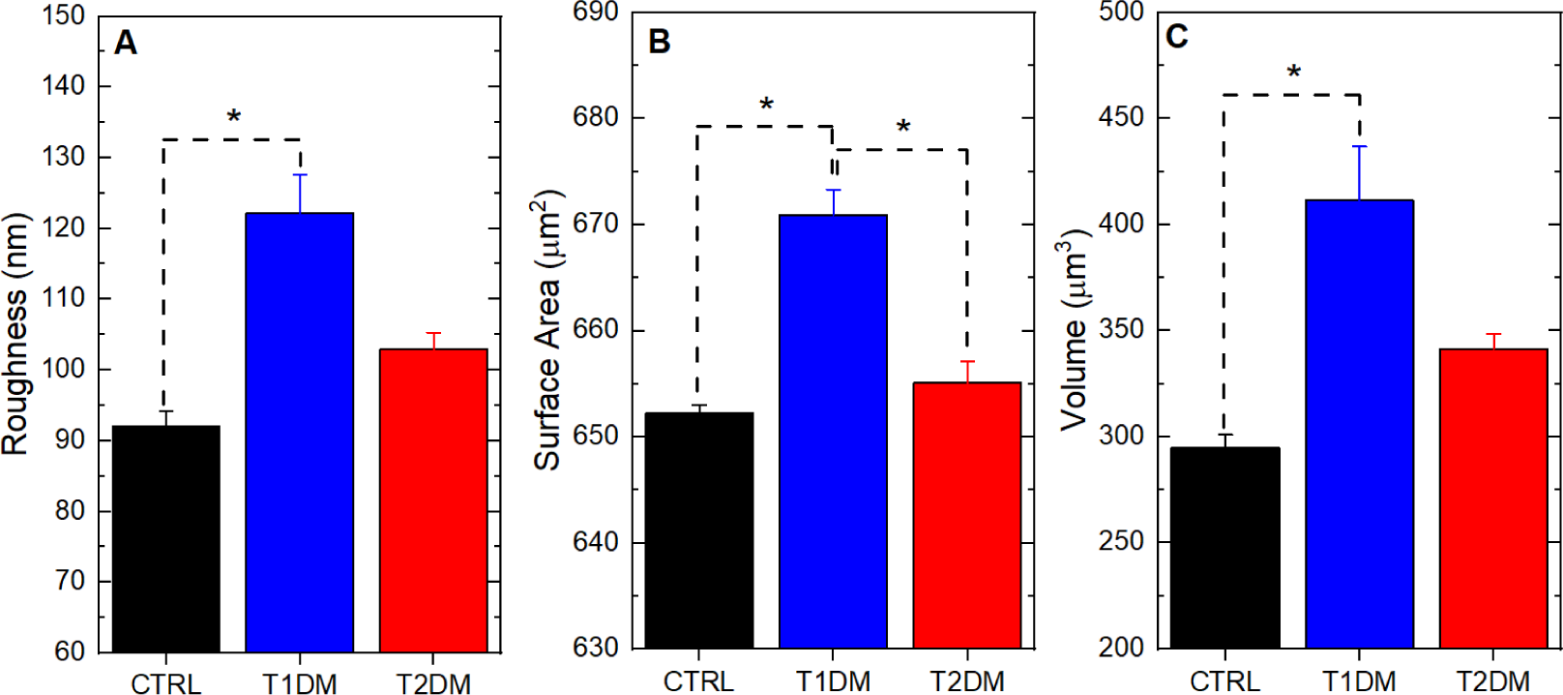
AFM quantitative data. Quantitative data on ultrastructural properties of tissues from the control group (normoglycemic) and patients with diabetes, (A) roughness, (B) tissue surface area, (C) volume charts. Asterisks indicate significant differences in the ANOVA test with Turkey for *p* < 0.05.

The morphological analysis of the tissue motivated the study of quantitative ultrastructural parameters of the hippocampus, as shown in Figure 2. Figure 2A shows the mean square roughness results of the analyzed tissues. The R_q_ mean values and their respective standard errors are 91.9 ± 4.3 nm, 122.2 ± 10.8 nm, and 102.9 ± 4.7 nm for the control, T1DM, and T2DM tissues. Indeed, the average roughness is higher for tissue samples with Diabetes compared to the control sample. These surface roughness results have been identified as a new tissue surface biomarker and have been widely applied in the study of biological systems, including the characterization of materials and nanotechnology, combined with the high resolution of measurements via AFM.^56,57^

Two other important ultrastructural data that can be analyzed from AFM measurements are surface area and volume (Figure 2B,C). The Surface Area values and their respective standard error are 652.3 ± 1.6 μm^2^, 670.9 ± 4.8 μm^2^, and 655.1 ± 4.1 μm^2^ for the control, T1DM, and T2DM tissues. One can observe an increase in the average value of the area for the groups with Diabetes concerning the control sample. Figure 2C shows the volume results of the analyzed tissues, showing the increase in volume for tissues with T1DM and T2DM. The volume values and their respective standard error are 295.1 ± 11.7 μm^3^, 411.4 ± 50.1 μm^3^, and 341.5 ± 13.8 μm^3^ for the control, T1DM, and T2DM tissues.

### Nanomechanical Results of Hippocampus Analysis

**Figure 3:**
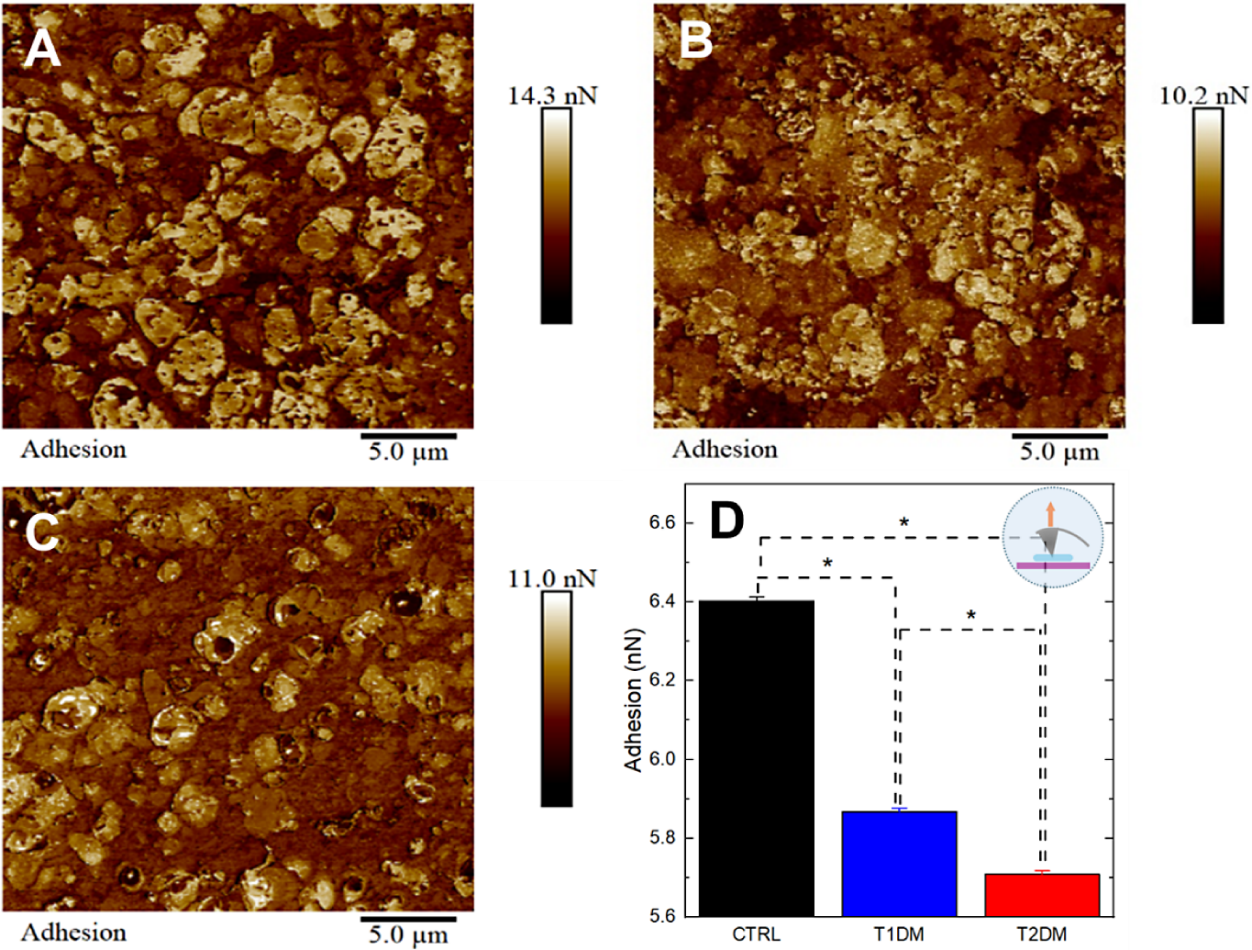
AFM adhesion data. Qualitative and quantitative adhesion data of tissues from the control group and DM subjects. (A) Adhesion map of control, (B) T1DM, and (C) T2DM group. (D) Bar chart plot of adhesion forces comparing the different groups. Asterisks indicate significant differences in the ANOVA test with Turkey for *p* < 0.05.

The characteristic adhesion maps of each group are shown in Figure 3A–C. The study of adhesion via AFM in biological tissues is highly relevant for understanding cellular and molecular processes, such as protein conformation and specific protein interactions, and inferring the distribution of charges on the surface under investigation.^58^ The average values of the adhesion force, calculated from the retraction curves in the AFM (mean ± SE), are 6.4 ± 0.2 nN, 5.9 ± 0.2 nN, and 5.7 ± 0.1 nN for the control, DM1 and DM2 tissues (Figure 3D). Interestingly, the control sample has a higher average adhesion value than the T1DM and T2DM samples, following a decreasing trend in the control, T1DM, and T2DM groups. This suggestion points to changes in electronegativity between samples due to differences in dipole moments or charge distributions,^59^ which could be attributed to protein changes in the ECM observed in Diabetes.

**Figure 4:**
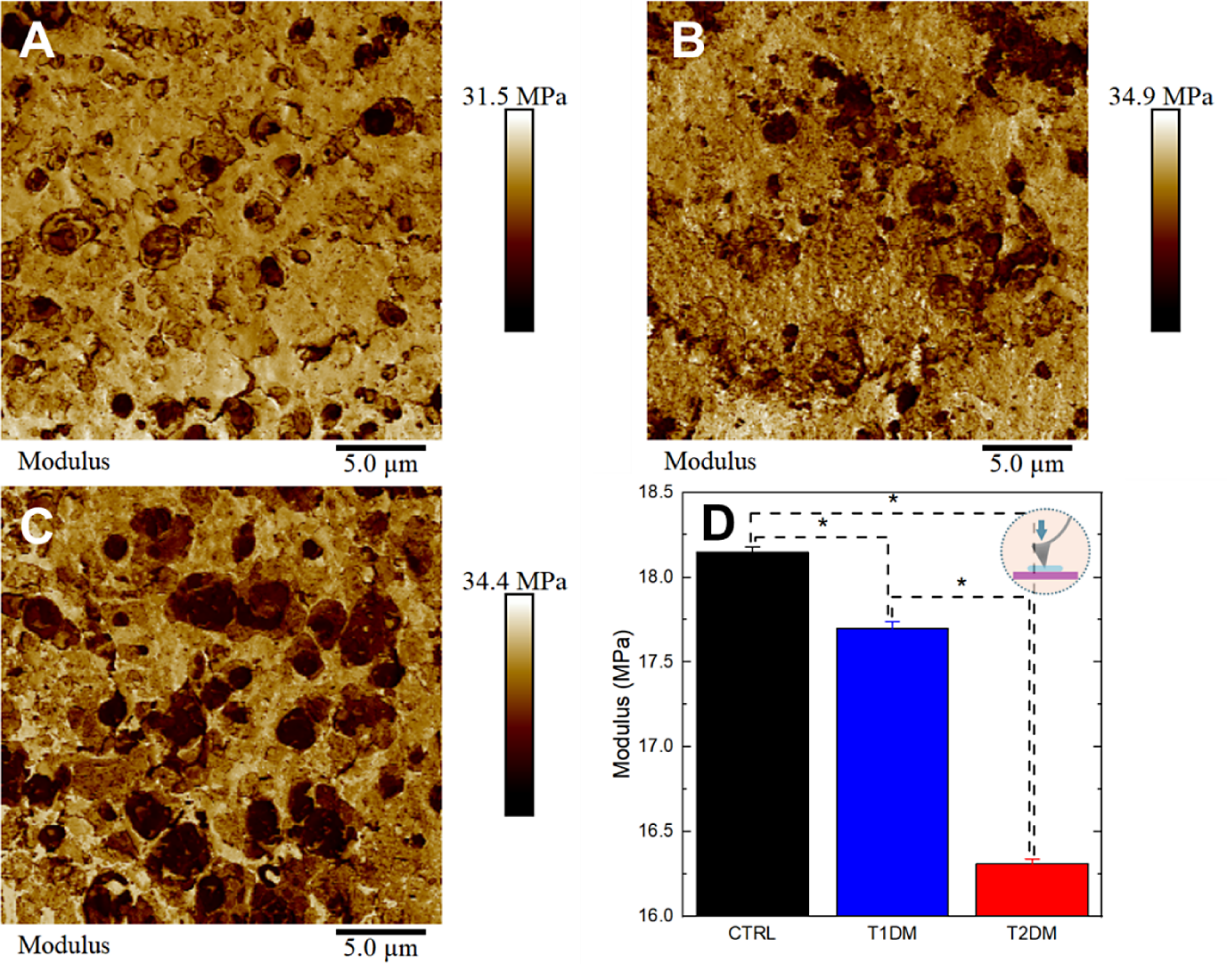
AFM modulus data. Qualitative and quantitative Young’s modulus (YM) data of tissues from the control group and DM subjects. (A) YM map of control, (B) T1DM, and (C) T2DM group. (D) Bar chart plot of YM comparing the different groups. Asterisks indicate significant differences in the ANOVA test with Turkey for *p* < 0.05.

Another important feature that can be analyzed via AFM is Young’s modulus (YM), also known as the modulus of elasticity. A high YM value indicates that the material is rigid and does not deform easily under stress. Characteristic maps of YM in the hippocampus are shown in Figure 4A–C. The YM values and their respective standard error are 18.5 ± 0.6 MPa, 17.6 ± 0.5 MPa, and 16,5 ± 0.6 MPa for the control, T1DM, and T2DM tissues (Figure 4D). Although the difference in means between the groups appears visually subtle, statistical analysis revealed a significant difference using the normality test (Turkey) followed by one-way ANOVA. This result suggests that the group variation was sufficiently small for the difference in means to become significant.

### Vibrational Results of Hippocampus Analysis

**Figure 5:**
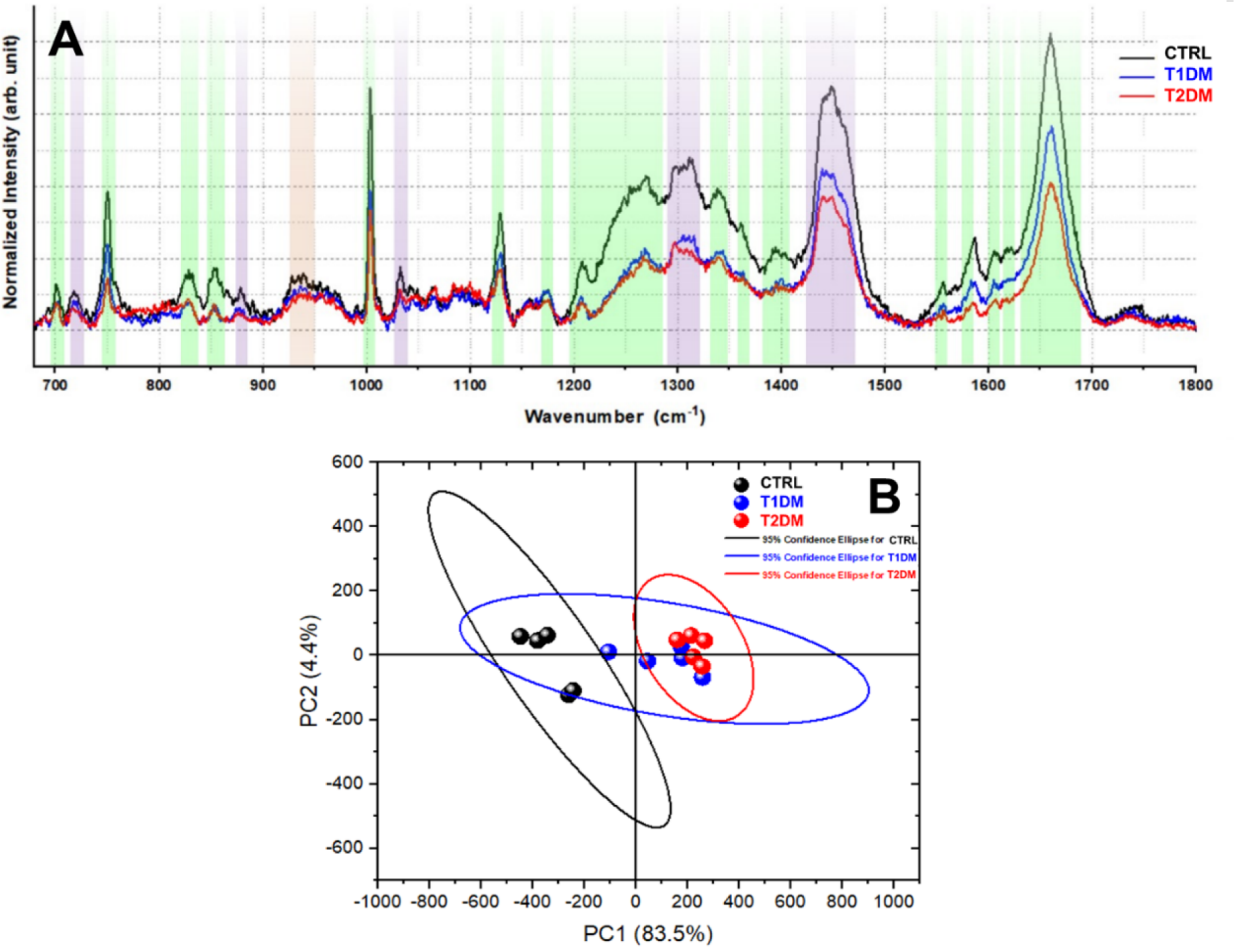
Molecular identification. A. Average spectra and identification of modes related to the control group (black), T1DM group (blue), and T2DM group (red). B. PCA analysis showing the differentiation between the control group (black), T1DM group (blue), and T2DM group (red), with a total variance of 87.9%.

Figure 5A shows the average spectra of the control, T1DM, and T2DM groups. It is possible to identify vibrational modes of the fundamental biochemical components of tissue structures: lipids and proteins. In the spectral region from 700 to 1800 cm^–1^, we can notice the presence of vibrational modes related to proteins, such as the Proline mode (855 cm^–1^), Tyrosine (831 cm^–1^, 1172 cm^–1^, 1609 cm^–1^), Phenylalanine (1004 and 1586 cm^–1^) and Tryptophan (750 cm^–1^, 1339 cm^–1^, 1362 cm^–1^, 1554 and 1619 cm^–1^). The presence of the Amide bands, in the range between 1207 and 1269 cm^–1^, attributed to Amide III and the mode at 1659 cm^–1^, related to Amide I, which is composed of carbon, oxygen, and nitrogen atoms (CONH), plays a crucial role in protein formation, forming crucial bonds that confer structural rigidity and provides information about the organization of secondary structure in actin filaments.^60^ Furthermore, bands corresponding to lipids were identified (718 cm^–1^, 880 cm^–1^, 1032 cm^–1^, 1307 and 1449 cm^–1^). These modes reflect the composition, organization, and structure of tissue lipids.

The exact wavelengths for each identified mode are listed in Table 1.

**Table 1:** Assignments of Each Peak of the Raman Spectrum^a^^,^^61−63^

Wavenumber (cm^–1^)	Amino acid/Protein	Lipid/Carbohydrate	Other
700	Methionine		
718		Choline	
750	Tryptophan		
831	Tyrosine		
855	Proline/Tyrosine		
	880	Sphingomyelin	
940			Polysaccharides
1004	Phenylalanine		
1032		CH_2/_CH_3_ben.	
1128	C – N str.		
1172	Tyrosine		
1207–1269	Amide III		
1307		CH_2_/CH_3_twi.	
1339	Tryptophan		
1362	Tryptophan		
1405	CH def.		
1449		C – H vib.	
1554	Tryptophan		
1586	Phenylalanine		
1609	Tyrosine		
1619	Tryptophan		
1659	Amide I		

a Abbreviation: str. = stretching, vib = vibration, ben = bending, twi = twisting, def = deformation.

Using multivariate Principal Component Analysis (PCA), it was possible to identify changes in vibrational modes that clearly distinguish between the control and hyperglycemic groups (T1DM and T2DM), as shown in Figure 5B. Analysis of the spectra by PCA reveals a significant distinction between the control and hyperglycemic groups (T1DM and T2DM). The main variations responsible for 87.9% of the variation in PCA are related to the intensities of the vibrational modes around 1004 cm^–1^, 1207–1269 cm^–1^, 1449 cm^–1^, and 1659 cm^–1^. These associated modes are lipid components (1449 cm-^–1^), the Amide group (1207–1269 cm^–1^ and 1659 cm^–1^), and phenylalanine (1004 cm^–1^), according to Table 1, and were all decreased in diabetes samples, suggesting a global disarrangement of the hippocampal vibrational signature of these animals.

## Discussion

Both T1DM and T2DM are known to lead to cognitive impairment through hippocampal damage.^64,65^ Here, we show fundamental biophysical alterations using cryosection of the hippocampus of T1DM and T2DM rats. Indeed, both diabetic rat models had impaired cognition associated with increased hippocampal roughness, volume, surface area, and reduced adhesion and YM forces. These alterations seemed more prominent in T1DM rats, indicating more significant hippocampal damage. Overall, these biophysical alterations were linked to a global disarrangement of these tissues, as evidenced by Raman Spectroscopy. These findings shed light on the biophysical aspects of how Diabetes hinders the hippocampus and could improve our understanding of diabetes-associated cognitive decline.

Figure S2 shows that T1DM is associated with atrophy of adipose and muscle tissue, while T2DM is characterized by increased mass due to fat accumulation. In T1DM, insulin production is very low or nonexistent, resulting in high blood glucose levels. In T2DM, insulin resistance leads the pancreas to produce more insulin, increasing its levels in the blood. Both types are marked by hyperglycemia and high glucose intolerance, with diabetic animals showing larger areas under the glucose tolerance test (GTT) curve. Figure S3 generally reveals that T1DM increases organ mass due to inflammation and edema. It also shows that diabetes affects perception, causing fear and difficulty distinguishing objects. Diabetic animals explored a new object less than controls, indicating memory deficits. Figure S4 shows that in novel object recognition tests, both diabetic groups had reduced exploration times, suggesting anxious behavior and deficits in episodic memory. Additionally, diabetic animals had difficulty learning the escape platform’s location and recalling its previous quadrant, indicating deficits in hippocampus-dependent learning and memory consolidation. These findings suggest that diabetes impacts cognition, with anxious behavior and impaired memory, comparable to earlier studies on cognitive deficits in diabetes and aging models. More details on all these results can be found in the Supporting Information.

T1DM and T2DM are known to lead to cognitive impairment through hippocampal damage, according to results in Figure S4. We highlight that in T1DM, cognitive deficits are frequently observed in executive functions, attention, and working memory. Frequent episodes of hypoglycemia can cause neuronal damage and reduce hippocampal synaptic plasticity, compromising learning and memory.^66^ In contrast, T2DM is associated with a higher risk of progressive cognitive decline and dementia, including Alzheimer’s. Patients with T2DM often experience difficulties in episodic memory, executive functions, and processing speed. Insulin resistance, chronic inflammation, and cardiovascular comorbidities contribute to cognitive impairment and increase the risk of neurodegeneration.^15^

In the morphological profile of the control group, structures internal to the pores are not seen in the samples from the TDM groups (blue arrows). In Figure 1, numerous holes can be seen. The most striking changes in the morphological profile observed may be related to the redistribution of proteins and local cellular structural rearrangement arising from hyperglycemic complications, as also observed in the results of the diameter and depth of the holes in the hyperglycemic tissues in relation to the control tissue. Indeed, Magariños and collaborators showed morphological changes in STZ-induced T1DM, including dendritic atrophy in pyramidal neurons, neuronal synaptic reorganization, and increased proliferation of astrocytes.^67^ Furthermore, this same study concluded that there was a redistribution of synaptic proteins in the tissue, which could indicate the interruption of the formation of continuous synapses between neurons in the hippocampus of an animal model with Diabetes induced by streptozotocin.^68^

Regarding T2DM, data from the study by Wrighten and colleagues^69^ indicate that cognitive decline due to T2DM may be partially attributable to structural rearrangement and changes in the electrophysiological properties of hippocampal neurons. At the same time, a study by Park and collaborators^70^ showed that brain tissue affected by Alzheimer’s Disease had enlarged holes with irregular distribution, unlike healthy tissue. These studies corroborate our morphology results and converge with the propositions highlighted here, showing that changes in the morphology of the diabetic hippocampus may be associated with cognitive dysfunctions, such as memory and learning, according to the cognitive results in Figure S4, which show that hyperglycemic animals had negatively affected memory and learning capacity.

In line with morphological changes, we found increased roughness in diabetic samples, shown in Figure 2A. These could be attributed to the accumulation of free fatty acids, advanced glycation products (AGE), and/or excess cytokines and neurotoxins on the surface of the hippocampus. It is important to emphasize that the hippocampus, a brain region crucial for cognition, is highly sensitive to insulin and has a high expression of receptors for this hormone.^71^ This sensitivity is primarily due to insulin’s direct involvement in regulating neurogenesis, synaptogenesis, and synaptic plasticity.^72^ Indeed, conditions of hypoinsulinemia, such as those observed in T1DM, lead to reduced long-term potentiation,^73^ diminished synaptic transmission,^73^ and progressive degeneration and death of hippocampal neurons,^74^ resulting in severe cognitive impairments.^73^ Conversely, conditions of hyperinsulinemia, associated with diminished insulin responsiveness—a context distinct from T1DM and characteristic of T2DM—also cause similar neuronal damage^75^ and functional impairments.^76,77^ This highlights that any alterations in insulin levels and response are detrimental to hippocampal homeostasis. The hippocampus is a key site for synaptic plasticity and memory formation. Chronic hyperglycemia and lack of glycemic control can lead to neuronal dysfunction and cell death in the hippocampus, affecting its structure and, possibly, surface roughness.^78^ It is also noteworthy that roughness is a nanometer measurement of height, which makes it plausible to associate these data with protein and molecular changes in the membrane. Again, attention is drawn to T1DM samples, which obtained the highest average roughness value compared to the other groups. Interestingly, Park and collaborators^70^ found increased roughness for Alzheimer’s Disease brain tissue compared to control brains. Therefore, the increased roughness herein identified in the diabetic hippocampus could be linked to neuroinflammation and oxidative stress similar to what was found in an Alzheimer’s Disease study.

It is important to note that changes in hippocampal roughness in Diabetes and Alzheimer’s Disease can be subtle and require sensitive analysis techniques, such as Atomic Force Microscopy, which is used in this research, to be detected and quantified. Although there are some similarities in the consequences of the two pathological conditions on the hippocampus, the underlying mechanisms are different. Alzheimer’s Disease involves specific neurodegenerative processes, while Diabetes Mellitus is a metabolic condition that can affect the brain more indirectly.

Under normoglycemic conditions, diabetes is not generally associated with increased hippocampal surface area, as shown in Figure 2B. On the contrary, Diabetes is more often associated with adverse effects on the nervous system, such as brain atrophy, neuron damage, and other harmful changes in the brain’s structure, including the hippocampus. However, the scan size used in this research and the interpretation of the results should be highlighted again, considering we are examining cellular structures within the tissue. Therefore, to elucidate the area data presented above, we observed that, in some cases, Diabetes can cause fluid retention and cerebral edema. This can temporarily increase brain volume, including the hippocampus. Cerebral edema may also result in a local increase in hippocampal surface area.^79^ A study by Song and collaborators^80^ showed that the brain water content of hyperglycemic animals with cerebral edema increased compared to the normoglycemic group, which agrees with the pathway proposed here. In this sense, Yuen et al.^81^ demonstrated in their study that rats with STZ-induced T1DM presented a phenotype similar to that of our animals, in addition to diabetic ketoacidosis, cerebral hypoperfusion, and edema. Thus, it is reasonable to suggest that our animals probably presented the same disorders, corroborating the volume of data presented. Again, as reported in the morphology results, a change was observed between the diabetic and control groups. Chronic hyperglycemia, characteristic of Diabetes, can cause oxidative damage and inflammation in the brain, affecting the integrity of neuronal cells and hippocampal structures. This fact may contribute to changes in surface area.

The main differences between T1DM and T2DM in terms of structural changes in the hippocampus are related to the magnitude and underlying mechanisms of these alterations. In T1DM, the greater roughness, volume, and area of the hippocampus can be attributed to more aggressive compensatory responses to repeated episodes of abnormal glycemia. In contrast, in T2DM, hippocampal remodeling is less pronounced due to insulin resistance and chronic inflammation, resulting in a less significant structural increase. These differences highlight the need for distinct therapeutic approaches to protect brain health in patients with T1DM and T2DM.^82^

In force spectroscopy measurements via AFM, adhesion forces can be associated with electrostatic, van der Waals, capillary forces, and forces promoted by breaking chemical bonds.^83^ In nonfunctionalized probes (like the ones we used), the adhesion forces are taken as nonspecific interactions, and it is impossible to separate the contribution of each of these forces. However, as the probes used here to analyze all samples were made of the same material and have the same specifications (model, tip radius, etc.), as well as the experiments for all samples were carried out under the same conditions (temperature and humidity air), differences in adhesion forces between samples may mean changes in their physical properties resulting from Diabetes, indicating changes in the sample surface.^84^ Another striking feature of AFM measurements performed in QNM mode^25^ is the high frequency of force curve acquisition, enabling a high-resolution map with 65 536 force curves. This high curve acquisition rate can induce air friction electrification in the AFM probes. Thus, it is consistently observed in our measurements that samples known to have positive charges have more significant adhesion interaction with the AFM probe in this mode. The opposite is observed for samples with a negative charge.^23^

Differences in adhesion strength observed in the hippocampus with T1DM and T2DM compared to the healthy hippocampus, when analyzed via AFM, can be attributed to several complex causes and mechanisms that may be related to the microstructural and biochemical changes resulting from Diabetes, such as changes in the Extracellular Matrix (ECM).^85^ Diabetes can lead to changes in the composition and organization of the ECM, a complex network of proteins and glycoproteins surrounding cells in brain tissue. These changes can affect the adhesion of cells and AFM probes to the diabetic hippocampus. Diabetes is associated with the accumulation of abnormal proteins, such as β-amyloid aggregation,^86^ which can affect neural cells’ mechanical properties and adhesion. Taken together, changes in the ECM or accumulation of aggregated proteins could explain adhesion changes found in the diabetic hippocampus, although other components cannot be excluded.

A higher mean YM value is observed for control tissues compared to T1DM and T2DM tissues, as shown in Figure 4D. This can be attributed to microstructural and biomechanical changes due to Diabetes, as mentioned above. These include changes in ECM deposition, protein aggregation, inflammation, and oxidative stress. For instance, changes in the ECM can reduce the elasticity of the diabetic hippocampal tissue since the cell tends to reorganize itself in the face of changes caused by pathology.^87^ In addition to these factors, cellular swelling observed by increased hippocampal tissue volume might also be associated with changes in YM. Altogether, both T1DM and T2DM profusely change the biophysics of hippocampal tissue, decreasing YM and adhesion while increasing roughness and volume–all of which seem to be associated with cognitive decline.

Viscoelastic materials exhibit varying responses to different perturbations. In PeakForce QNM, the cantilever oscillates at frequencies in the kHz range, which induces different elastic behaviors at each frequency.^88^ It is important to note that AFM measurements at higher scan frequencies reveal more elastic properties, leading to higher elastic modulus values.^89^ As the indentation frequency increases, the viscoelastic material’s response becomes stiffer.^88−90^ Since the QNM mode acquires force curves at frequencies around 1 kHz, the tissue appears to have a higher modulus, particularly in high-resolution maps.^25^ This must be considered when interpreting results, as viscoelasticity can introduce variability. However, despite the higher absolute values, the relative differences between control and hyperglycemic tissues remain valid and accurately reflect mechanical changes due to hyperglycemia, given that all measurements were conducted under consistent experimental conditions. It is important to highlight that Young’s Modulus values shown here are higher than expected for these types of tissues. This may be due to a combination of factors, such as measurements at high frequencies (in the order of kHz) and experiments performed in the air environment.

Regarding the vibrational results obtained using Raman spectroscopy, in Figure 5, a marked decrease was observed for several peaks in hyperglycemic tissues compared to those of control tissues. The individual spectra show that the most significant changes are in the amide, cholesterol, and lipid bands. This suggests fundamental molecular alterations in the hippocampus upon Diabetes, which are associated with changes in lipids and proteins, again showing the structural global changes that the tissue presents, which can be attributed to complications arising from hyperglycemia. It is, therefore, noted how Diabetes, a disease characterized by metabolic disorders, can cause such changes by affecting a tissue that plays a fundamental role in producing synapses, memory, learning, and cognition.

These results show how altering different substances affects the functioning and morphology of the hippocampus under diabetic conditions. Methionine, for example, plays various roles in the body, including synthesizing proteins and neurotransmitters.^91^ Reduced levels may indicate cellular dysfunctions resulting from Diabetes, affecting memory and learning, as evidenced by the peak reduction at 718 cm^–1^. Tryptophan (bands at 750 cm^–1^, 1339 cm^–1^, 1362 cm^–1^, 1554 cm^–1^, and 1619 cm^–1^) and phenylalanine (band at 1004 cm-1), precursors of neurotransmitters such as serotonin and dopamine, are shown to be reduced in diabetic hippocampal tissues, correlating with symptoms of depression and anxiety.^92^ Tyrosine (bands at 831 cm^–1^, 1172 cm^–1^, and 1609 cm^–1^), another amino acid, is also affected, suggesting cognitive complications and mood changes.^93^ Furthermore, lipids such as sphingomyelin (band at 880 cm^–1^), essential for cellular integrity, and polysaccharides, which modulate the inflammatory response, show alterations in diabetic tissues, contributing to neuronal and morphological dysfunctions.^94^ Changes in the amide bands and C–H vibrations (band at 1449 cm^–1^) observed in diabetic tissues indicate alterations in the structure and molecular interactions, highlighting the direct impact of Diabetes on brain function.^95^

The decrease in Raman peaks, in general, in hippocampal tissue components may be related to morphological changes caused by Diabetes. These peaks represent the molecular properties of tissues, including their chemical composition and molecular structure. A decrease in the intensity of these peaks may indicate changes in the structural integrity of brain tissue, such as reduced cell density, loss of neuronal connections, or even degeneration of nerve cells. In Diabetes, hyperglycemia can trigger oxidative stress and chronic inflammation, known to cause tissue damage, including the hippocampus, as highlighted in the previous results. This damage can lead to morphological changes, such as neuronal atrophy, loss of neurons, or changes in cellular architecture. As a result, the decrease in Raman peaks may reflect these morphological changes, providing important information about the effects of Diabetes on the structure and health of brain tissue.

The data presented here corroborate the AFM data. While Raman spectroscopy provided information on changes in biochemical composition, AFM provided complementary data on changes in the tissue’s mechanical properties. Together, these techniques reinforce the conclusion that hyperglycemia induces notable changes in tissue composition and mechanics, corroborating the findings.

## Conclusions

The results presented in this work demonstrated clear biophysical and vibrational impairments in the hippocampus of T1DM and T2DM rats. Diabetic animals had cognitive deficits associated with increased roughness, area, and volume and decreased adhesion and YM. We also found an overall reduction in the vibration signature of diabetic samples when compared to those of control tissue. The results presented in this work are fundamental for better understanding the relationship between hyperglycemia and cognitive impairment, providing biophysical cues of the hippocampus linked to cognitive decline in Diabetes.

## Supporting Information Available

Details of the methodology and results of the cognitive tests carried out, details of the results of the characterization of diabetes and cognition profile, details of force curve x distance in hippocampus (PDF)

## Notes

Ethical Committee on Animal Use and Welfare of the Federal University of Maranhão, under ruling number 23115.000747/2022–90.
