# Supplementary material for: Diabetes and Cognitive Decline: An Innovative Approach to Analyzing the Biophysical and Vibrational Properties of the Hippocampus
Source: ACS Omega. 2024 Sep 19;9(39):40870–81. doi: 10.1021/acsomega.4c05869 (PMC11447714; doi:10.1021/acsomega.4c05869)
Supplement: Supplementary file 1 — ao4c05869_si_001.pdf [file ao4c05869_si_001.pdf]

SUPPLEMENTARY MATERIAL

DIABETES AND COGNITIVE DECLINE: AN INNOVATIVE APPROACH TO ANALYZING THE BIOPHYSICAL AND VIBRATIONAL PROPERTIES OF THE HIPPOCAMPUS

Maria do Socorro do Nascimento Amorim[1,2], Erick Rafael Dias Rates[1], Isabela Vitoria de Araujo Costa Melo[1], Joel Félix Silva Diniz Filho[1], Clenilton Costa dos Santos[1], Ralph Santos-Oliveira[3,4], Renato Simões Gaspar[5], Jonas Rodrigues Sanches[6], Bruno Araújo Serra Pinto[6], Antonio Marcus de Andrade Paes[5] and Luciana Magalhães Rebelo Alencar[1*]

[1]Federal University of Maranhão, Department of Physics, Laboratory of Biophysics and Nanosystems, Campus Bacanga, São Luís, 65080-805, Maranhão, Brazil

[2] Federal University of Maranhão, University School, Campus Bacanga, São Luís, 65080-805, Maranhão, Brazil

[3]Brazilian Nuclear Energy Commission, Nuclear Engineering Institute, Rio de Janeiro 21941906, Brazil

[4]Rio de Janeiro State University, Laboratory of Nanoradiopharmacy, Rio de Janeiro 23070200, Brazil

[5]Campinas State University, Translational Medicine Department, Campinas, Sao Paulo, 13083888, Brazil

[6]Federal University of Maranhão, Department of Physiological Sciences, Laboratory of Experimental Physiology, Campus Bacanga, São Luís, 65080-805, Maranhão, Brazil

OVERVIEW

In the supplementary material, additional details that are not included in the main article due to space limits are provided below:

1. Experimental Design
2. Details of the methodology of the cognitive tests carried out (see Section 1).
3. Details of the results of the characterization of diabetes and cognition profile (see Section 2).
4. Discussion of results (see Section 3).
5. Curve Force x Distance

1 EXPERIMENTAL DESIGN

The experiment was conducted over 24 weeks and divided into five phases, as shown in Figure S1. In Phase I (weeks -6 to 0), female rats were fed a high-sucrose diet to induce obesity and type 2 diabetes (T1DM). Mating occurred in Phase II (week 0), and pregnant rats nursed their pups. In Phase III (week 12), the pups were divided into three groups: control (CTR) receiving saline, type 1 diabetes group (T1DM) induced by STZ (65 mg/kg), and T2DM group that continued to receive saline. In Phase IV (week 23), rats underwent cognitive tests like the water maze and object recognition tests. Finally, in Phase V (week 24), rats were euthanized, and blood and brain samples were collected for biochemical and morphometric analyses, including hippocampal analyses by AFM and Raman spectroscopy.

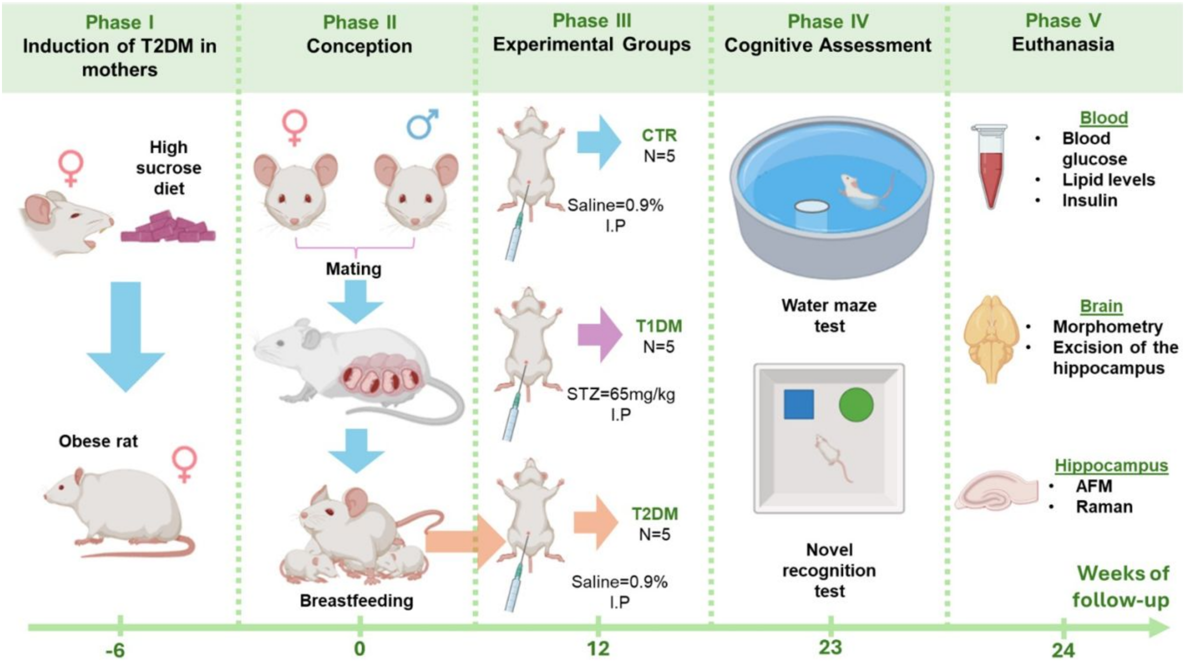

Figure S1: Experimental design to obtain control samples, type 1 and type 2 diabetes (T2DM) in rats. In Phase I, female rats were induced to T2DM using a high-sucrose diet. After mating and nursing (Phase II), the pups were divided into experimental groups (Phase III): control (CTR), STZ-induced T1DM, and T2DM. In Phase IV, the rats were subjected to cognitive tests. In Phase V, euthanasia was performed to collect blood and brain samples for biochemical and structural analyses of the hippocampus.

2 COGNITION TESTS

The animals underwent a water maze test to assess hippocampus-dependent spatial learning and memory [1]. The apparatus comprised a circular fiberglass pool filled with water (22 ºC), partitioned into four quadrants, and featuring visible cues on the walls for orientation.

A hidden escape platform was positioned 1.5 cm beneath the water surface in one pre-set quadrant. Over three consecutive days, the animals underwent four trials per day. They were individually released into the water from one of the four quadrants, facing a maze wall, and given 60 seconds to locate the hidden platform. Rats unable to find the platform within this time were gently guided to it and allowed to remain there for 30 seconds. The latency to find the hidden platform was recorded as an indicator of spatial learning, expressed as the meantime across daily trials. On the fourth day, the platform was removed, and the animals were released into the pool's center to swim for 120 seconds. The time spent in the quadrant where the platform was previously located was recorded as a measure of short-term memory retention.

Concurrently, episodic memory was evaluated using the novel object recognition test [2], consisting of three stages: habituation, familiarization, and testing. During habituation, the animals were placed in a square box (2,500 cm^2), identical to the one used in subsequent stages, and allowed to explore for 5 minutes freely. After a 24-hour interval, the familiarization stage commenced. Two similar objects were positioned in opposite quadrants within the box. The animals were then individually introduced into the center of the box for 5 minutes of free exploration, during which the exploration time for each object was recorded. Following a 1-hour delay, the testing phase began. One of the objects was replaced with a novel one of a different shape. The animals were reintroduced into the box and given 5 minutes for exploration, with the exploration time for each object recorded. Episodic memory assessment was based on the time spent by the animal exploring the novel object relative to the total exploration time.

2 RESULTS FROM THE ASSESSMENT OF DIABETES AND COGNITION

Characterization of Diabetes

Initially, we determined the overall metabolic state of CTR, T1DM, and T2DM animals (Figure S2). Male offspring from mothers fed HSD (T2DM animals) exhibited greater body mass than CTR, while T1DM rats showed reduced body mass. Consistent with the body mass data, T1DM animals appeared lean (Figure S2C), exhibited sarcopenia (Figure S2D), and experienced a significant reduction in adipose mass (Figure S2E). Conversely, T2DM animals showed signs of central obesity (Figure S2C), obesogenic sarcopenia (Figure S2D), and accumulation of both visceral and non-visceral adipose tissue (Figure S2E).

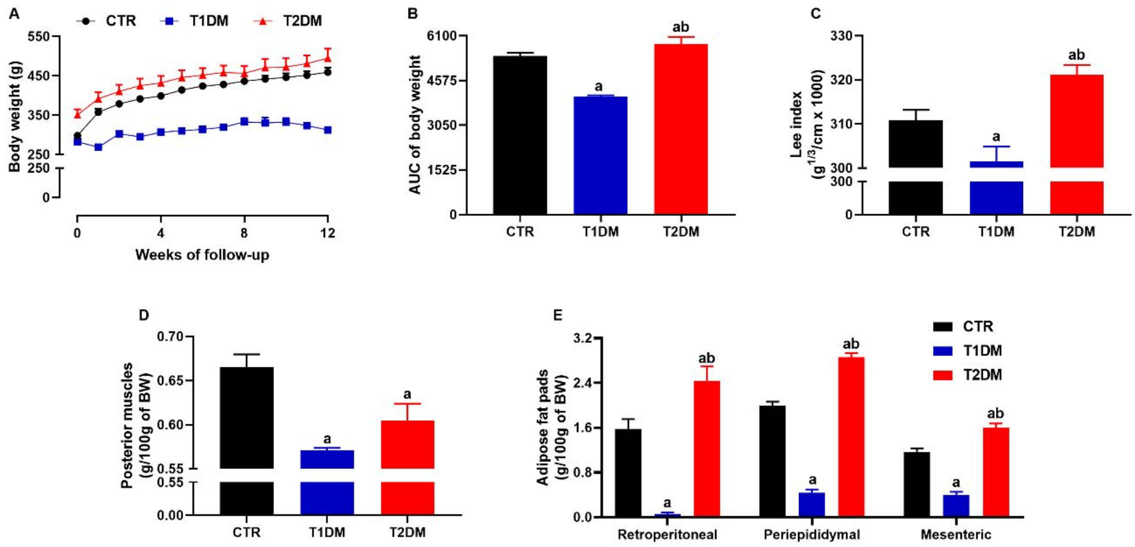

Figure S2. **Morphometric differences between types of Diabetes mellitus**. A, body weight (g); B, area under the curve (AUC) of body weight; C, Lee index ($g^{1/3}$/cm·1000); D, relative weight (g/100g of BW) of posterior muscles (soleus and gastrocnemius); E, relative weight (g/100g of BW) of retroperitoneal, periepididymal and mesenteric fat pads assessed in control rats (CTR, n=5), type 1 diabetic rats (T1DM, n=5) and type 2 diabetic rats (T2DM, n=5). Dots and bars represent mean ± SEM (One-way Anova-Tukey). ^{a} represents $p < 0.05$ when compared with CTR, while ^{b} represents $p < 0.05$ when compared to T1DM.

Regarding the glucose-insulin axis, the T1DM group presented severe fasting hyperglycemia (Figure S3A), elevated triglyceride levels (Figure S3B), glucose intolerance (Figure S3C-D), hypoinsulinemia (Figure S3E), and both peripheral (Figure S3F) and hepatic (Figure S3G) insulin resistance. While the T2DM group also displayed increased glycemic levels (Figure S3A), considerable hypertriglyceridemia (Figure 2B), and glucose intolerance (Figure S3C-D) similar to the T1DM group, it demonstrated an opposite pattern in terms of insulin levels, showing hyperinsulinemia (Figure S3E). Finally, the T2DM group ultimately exhibited degrees of peripheral (Figure S3F) and hepatic (Figure S3G) insulin resistance identical to those of the T1DM group. Our data show classical DM1 and DM2 features in the respective animal groups.

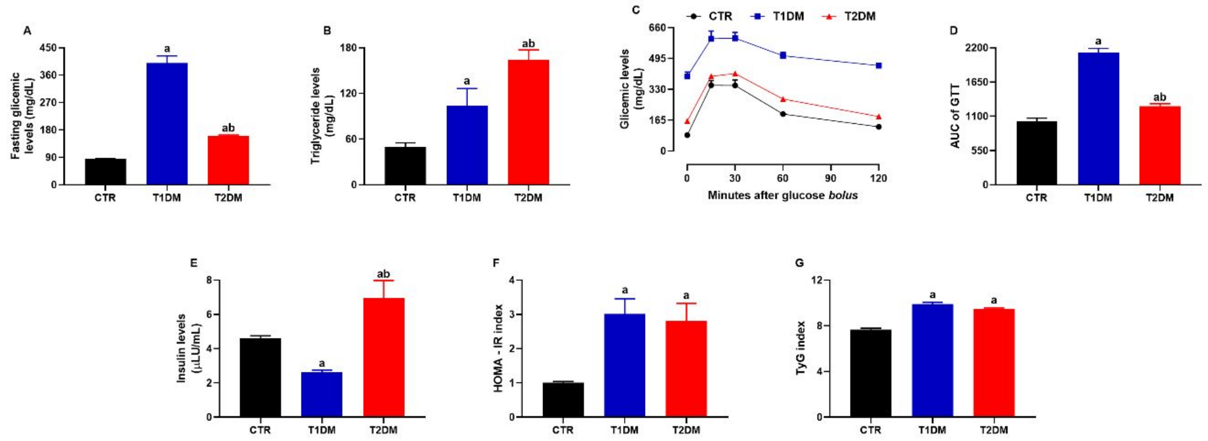

Figure S3. Glucose-insulin axis and insulin resistance in different types of Diabetes mellitus. A, Fasting glycemic levels (mg/dL); B, serum triglycerides levels (mg/dL); C, glycemic levels (mg/dL) during glucose tolerance test – GTT; D, AUC of glycemic levels during GTT; E, serum insulin levels (µLU/mL); F, HOMA index to establish peripheral insulin resistance; and G, TyG index to establish hepatic insulin resistance in control rats (CTR, n=5), type 1 diabetic rats (T1DM, n=5) and type 2 diabetic rats (T2DM, n=5). Dots and bars represent mean ± SEM (One-way Anova-Tukey). [a] represents $p < 0.05$ when compared with CTR, while [b] represents $p < 0.05$ when compared to T1DM.

Assessment of cognition

After confirming the induction of the different types of Diabetes, groups underwent cognitive performance assessment tests. Initially, regarding the morphometric evaluation of the collected brains, differences were observed only in the brains of the T1DM group, which were heavier (Figure S4A).

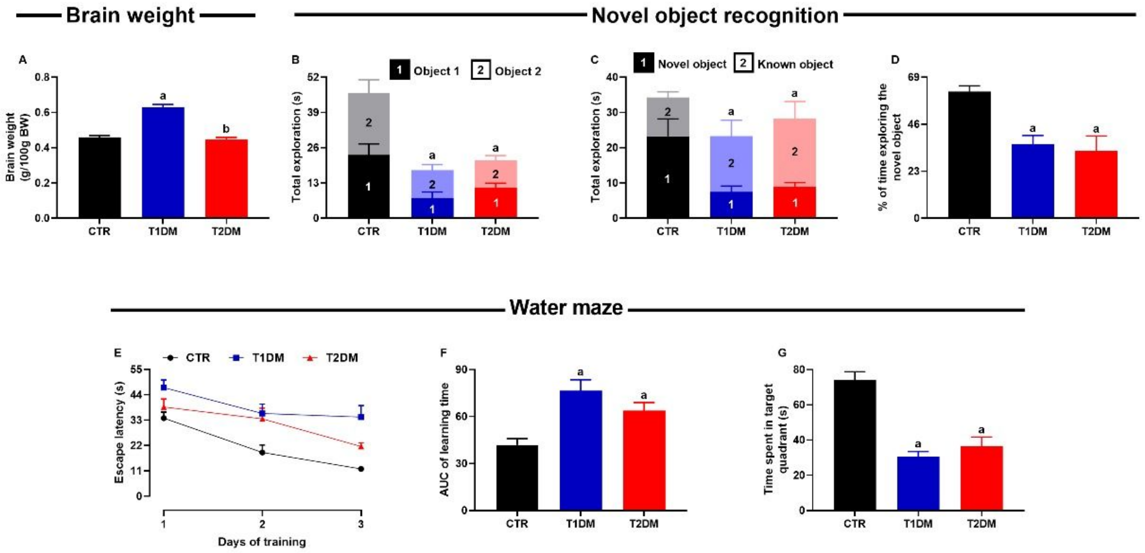

Figure S4. Brain morphometry and cognitive function in different types of diabetes mellitus. A, relative weight (g/100g of BW) of the total brain; B, total time (s) spent exploring the two identical objects during the familiarization stage in the novel object recognition test; C, total time (s) spent exploring both novel and familiar objects during the novel object recognition test; D, relative exploration time (%) of the new object in the novel object recognition test; E, time (s) spent learning the location of the escape platform on consecutive training days in the water maze test; F, AUC of learning time in water maze test; G, exploration time (s) to the target quadrant in the water maze test in control rats (CTR, n=5), type 1 diabetic rats (T1DM, n=5) and type 2 diabetic rats (T2DM, n=5). Dots and bars represent mean ± SEM (One-way Anova-Tukey). A represents $p < 0.05$ compared to CTR, while b represents $p < 0.05$ compared to T1DM.

3 DISCUSSIONS

The result in Figure S2A aligns with expectations since T1DM leads to atrophy in adipose and muscle tissue caused by mass loss. On the other hand, in T2DM, an accumulation of fatty tissue leads to mass gain and is associated with obesity [3]. This result is compatible with what was expected since, in T2DM, where insulin resistance is associated with receptor damage, not all receptors will be dysfunctional. There will still be glucose entry into the cell. In the case of T1DM, insulin secretion is null or very low and will not reach receptors satisfactorily, allowing a high glucose level to be free in the blood [4]. Figure S2E shows that T1DM is characterized by low production or absence of insulin, so the insulin dosage in the blood is lower than in control animals. In T2DM, as there is a defect in insulin receptors, the pancreas tends to produce more insulin as a compensation mechanism for signaling the entry

of glucose, causing more free insulin in the blood of these animals. Figure S2B shows that animals with T1DM and T2DM are hyperglycemic, which corroborates the characterization of diabetes so far. With the results in Figures S2C and S2D, we observed that animals with T1DM and T2DM show marked glucose intolerance compared to control animals due to low availability or resistance to insulin, which does not effectively allow glucose to enter cells. Figure S2D reinforces this result by showing that the area over the GTT curve for animals with T1DM is larger, showing their high glucose intolerance, followed by animals with T2DM.

About the result shown in Figure S3 A, it is noteworthy that the organs of T1DM animals had an increase in mass caused by inflammation and edema resulting from Diabetes. Figures S3 B and C show that Diabetes affects the diabetic animal's ability to perceive, highlighting that many diabetic animals do not even perceive objects in the area. It was also noted that many diabetic animals were afraid of the object inside the box, reinforcing the relationship between diabetes and anxiety [5]. It is also noteworthy that there is an approximately equal relationship between the interaction time with objects one and 2 for each group under study, showing a tendency to explore the two objects, which are initially unknown, equally. The animals interact with the new object when replacing one of the objects, as shown in Figure S3C. When looking at a new object, we observed in this graph that control animals explored it longer than animals with T1DM and T2DM. This result reveals that diabetic animals do not remember the characteristics of the old object and, therefore, tend not to explore the new object, as they do not distinguish between them. On the other hand, the control animals recognized a new object and dedicated more time to exploring what they did not yet know.

In contrast to the morphometric data, the cognitive repercussions associated with different types of Diabetes were similar. In the novel object recognition test, during the familiarization stage, both groups exhibited shorter exploration times of the two objects (Figure S4B). This could be interpreted as anxiety behavior [6]. Furthermore, in the test itself, both groups displayed a reduced interaction with the unfamiliar object (Figure S4C), indicating impairments in episodic memory independent of hippocampal function. Likewise, diabetic animals exhibited greater difficulty in learning the precise location of the escape platform based on environmental cues (Figure S4D-E), and they also failed to remember the quadrant where the platform had been previously located (Figure S4F). These findings suggest significant deficits in hippocampus-dependent learning and memory consolidation. Regardless of the model, diabetic rats had impaired cognition. For instance, many diabetic animals were afraid of the object inside the box, reinforcing the relationship between Diabetes and anxiety [5]. This is

comparable with previous studies. In the study by Mennenga and collaborators [7], animals treated with Harmine, a psicopharmac, showed motor and behavioral deficiencies, demonstrated by a lack of competence to perform the procedural components of the water maze test. In the study by Gardner and colleagues[8], the authors compared the effects of aging on learning several hippocampus-sensitive tasks, identifying impaired performance on an object location task for older rats.

4 CURVE FORCE X DISTANCE

Force-distance curves provide detailed information about the nanomechanical properties of tissues, such as stiffness and elasticity, which can be affected by pathological conditions [9]. In the curves presented in Figure S5, the blue line represents the approach curve, which shows the interaction between the AFM probe tip and the sample surface as the probe approaches the tissue. The red line represents the retraction curve, which reflects the behavior of the tissue when the probe is withdrawn.

The slope of the approach curve can provide information about the stiffness, calculated as Young's modulus, of the sample surface [10]. A steeper slope indicates a stiffer surface. Young's modulus values were extracted from fits to the force curves over a deflection range. The representative results for Control, T1DM, and T2DM samples were 20.1 MPa, 17.2 MPa, and 15.6 MPa, respectively. It is important to highlight that Young's Modulus values shown here are higher than expected for these types of tissues. This may be due to a combination of factors, such as measurements at high frequencies (in the order of kHz) and experiments performed in the air environment.

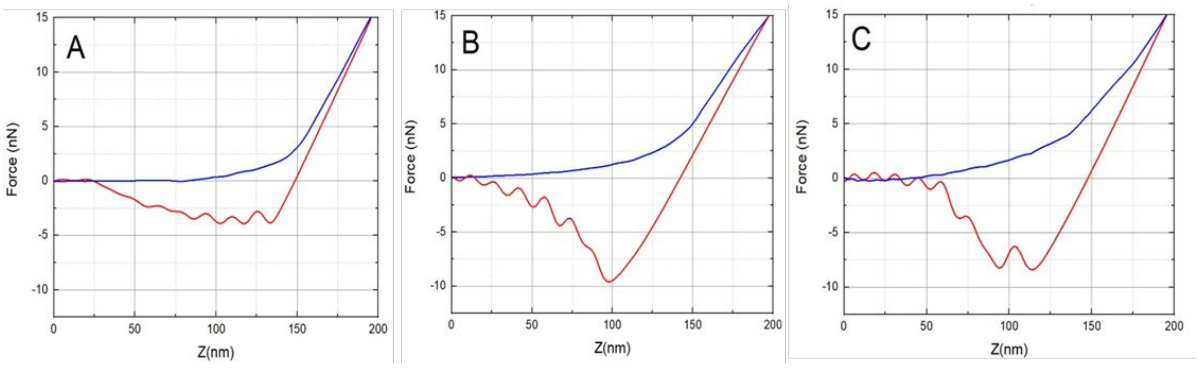

Figure S5: Examples of force-distance curves obtained by AFM for hippocampal samples. The blue curve is the approaching curve. The red curve is the retraction curve. A, control. B, Type 1 Diabetes Mellitus. C, Type 2 Diabetes Mellitus.

From the results shown in Figure S5, it can be observed that the hippocampal samples with T1DM and T2DM exhibit changes in stiffness compared to the control hippocampus, indicating that the pathological tissue is less resistant to deformation. This suggests that the tissue may deform more easily under pressure or stress, such as forces applied by the AFM probe in nanomechanical experiments. In a pathological context, such as in hippocampal tissues affected by diabetes or other neurodegenerative diseases, a decrease in Young's modulus may reflect structural changes, such as degradation of the extracellular matrix, loss of cellular integrity, or other alterations in tissue composition resulting in increased malleability, as mentioned in the main text.

The retraction curve is crucial for understanding tissue elasticity and adhesion [11]. In diabetic samples, the retraction curve typically shows greater deviations than the healthy hippocampus, suggesting changes in elastic recovery and possible alterations in cellular adhesion features that may be associated with the degenerative process caused by diabetes. These curves highlight the differences in mechanical behaviors between healthy tissues and those affected by diabetes, contributing to a better understanding of the microstructural changes and their implications for cognitive function.

REFERENCES

[1] Morris R. Developments of a water-maze procedure for studying spatial learning in the rat. J Neurosci Methods 1984;11:47–60. https://doi.org/10.1016/0165-0270(84)90007-4.
[2] Ennaceur A, Meliani K. A new one-trial test for neurobiological studies of memory in rats. III. Spatial vs. non-spatial working memory. Behav Brain Res 1992;51:83–92. https://doi.org/10.1016/s0166-4328(05)80315-8.
[3] Sala D, Zorzano A. Differential control of muscle mass in type 1 and type 2 diabetes mellitus. Cell Mol Life Sci 2015;72:3803–17. https://doi.org/10.1007/s00018-015-1954-7.
[4] Shields BM, Peters JL, Cooper C, Lowe J, Knight BA, Powell RJ, et al. Can clinical features be used to differentiate type 1 from type 2 diabetes? A systematic review of the literature. BMJ Open 2015;5:e009088. https://doi.org/10.1136/bmjopen-2015-009088.
[5] Smith KJ, Deschênes SS, Schmitz N. Investigating the longitudinal association between diabetes and anxiety: a systematic review and meta-analysis. Diabet Med 2018;35:677–93. https://doi.org/10.1111/dme.13606.
[6] Karpha K, Biswas J, Nath S, Dhali A, Sarkhel S, Dhali GK. Factors affecting depression and anxiety in diabetic patients: A cross sectional study from a tertiary care hospital in Eastern India. Ann Med Surg (Lond) 2022;84:104945. https://doi.org/10.1016/j.amsu.2022.104945.
[7] Mennenga SE, Gerson JE, Dunckley T, Bimonte-Nelson HA. Harmine treatment enhances short-term memory in old rats: Dissociation of cognition and the ability to perform the procedural requirements of maze testing. Physiol Behav 2015;138:260–5. https://doi.org/10.1016/j.physbeh.2014.09.001.

[8] Gardner RS, Newman LA, Mohler EG, Tunur T, Gold PE, Korol DL. Aging is not equal across memory systems. Neurobiol Learn Mem 2020;172:107232. https://doi.org/10.1016/j.nlm.2020.107232.
[9] Garcia R. Nanomechanical mapping of soft materials with the atomic force microscope: methods, theory and applications. Chem Soc Rev 2020;49:5850–84. https://doi.org/10.1039/D0CS00318B.
[10] Song Y, Bhushan B. Atomic force microscopy dynamic modes: modeling and applications. Journal of Physics Condensed Matter 2008;20:225012. https://doi.org/10.1088/0953-8984/20/22/225012.
[11] Heath GR, Scheuring S. Advances in high-speed atomic force microscopy (HS-AFM) reveal dynamics of transmembrane channels and transporters. Curr Opin Struct Biol 2019;57:93–102. https://doi.org/10.1016/j.sbi.2019.02.008.
